# Supplementary material for: Group I Metabotropic Glutamate Receptors Modulate Motility and Enteric Neural Activity in the Mouse Colon
Source: Biomolecules. 2023 Jan 9;13(1):139. doi: 10.3390/biom13010139 (PMC9856182; doi:10.3390/biom13010139)
Supplement: Supplementary file 1 [file biomolecules-13-00139-s001.zip › biomolecules-2074568-supplementary-done.pdf]

biomolecules

MDPI

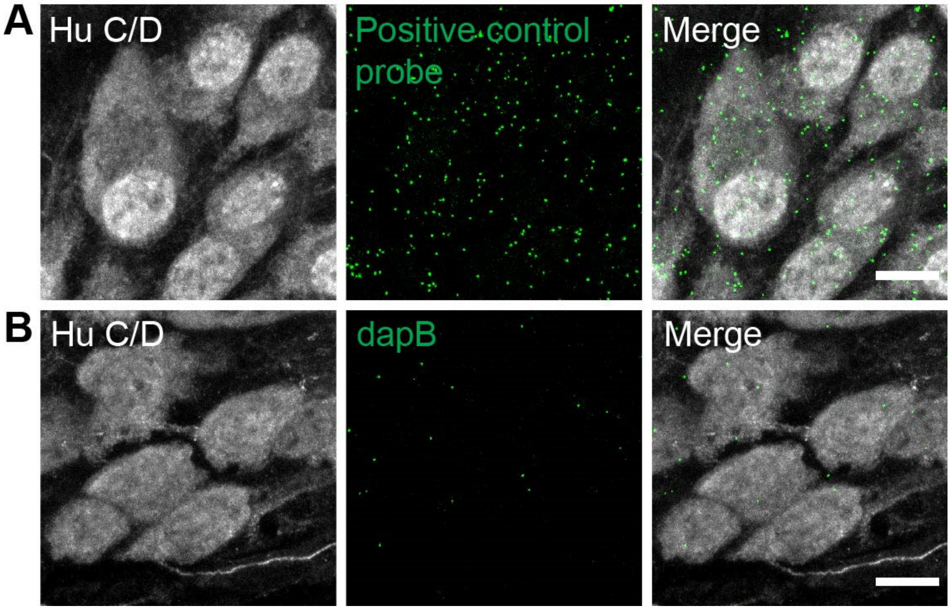

Figure S1: RNAscope controls. **(A)** Panels showing confocal micrographs of Hu C/D+ neurons (grey) and positive control probe (green). **(B)** Panels showing confocal micrographs of Hu C/D+ neurons (grey) and negative control probe dapB (green). Scalebars = 10µm.
